# *Sargassum horneri* methanol extract rescues C2C12 murine skeletal muscle cells from oxidative stress-induced cytotoxicity through Nrf2-mediated upregulation of heme oxygenase-1

**DOI:** 10.1186/s12906-015-0538-2

**Published:** 2015-02-05

**Authors:** Ji Sook Kang, Il-Whan Choi, Min Ho Han, Su Hyun Hong, Sung Ok Kim, Gi-Young Kim, Hye Jin Hwang, Byung Woo Kim, Byung Tae Choi, Cheol Min Kim, Yung Hyun Choi

**Affiliations:** Blue-Bio Industry RIC, Dongeui University, Busan, 614-714 Republic of Korea; Department of Microbiology, College of Medicine, Inje University, Busan, 608-737 Republic of Korea; Anti-Aging Research Center, Dongeui University, Busan, 614-714 Republic of Korea; Department of Biochemistry, Dongeui University College of Korean Medicine, Busan, 614-052 Republic of Korea; Team for Scientification of Korean Medical Intervention (BK21 Plus) and Department of Herbal Pharmacology, College of Korean Medicine, Daegu Haany University, Daegu, 706-828 Republic of Korea; Laboratory of Immunobiology, Department of Marine Life Sciences, Jeju National University, Jeju, 690-756 Republic of Korea; Department of Food and Nutrition, College of Natural Sciences & Human Ecology, Dongeui University, Busan, 614-714 Republic of Korea; Department of Life Science and Biotechnology, College of Natural Sciences & Human Ecology, Dongeui University, Busan, 614-714 Republic of Korea; Division of Meridian and Structural Medicine, School of Korean Medicine, Pusan National University, Yangsan, 626-870 Republic of Korea; Department of Biochemistry, Busan National University College of Medicine, Yangsan, 626-870 Republic of Korea

**Keywords:** *Sargassum horneri*, Oxidative stress, ROS, Apoptosis, Nrf2/HO-1

## Abstract

**Background:**

*Sargassum horneri*, an edible marine brown alga, is typically distributed along the coastal seas of Korea and Japan. Although several studies have demonstrated the anti-oxidative activity of this alga, the regulatory mechanisms have not yet been defined. The aim of the present study was to examine the cytoprotective effects of *S. horneri* against oxidative stress-induced cell damage in C2C12 myoblasts.

**Methods:**

We demonstrated the anti-oxidative effects of a methanol extract of *S. horneri* (SHME) in a hydrogen peroxide (H_2_O_2_)-stimulated C2C12 myoblast model. Cytotoxicity was determined using the 3-(4,5-dimetylthiazol-2-yl)-2,5-diphenyl-tetrazolium assay and mode of cell death by cell cycle analysis. DNA damage was measured using a comet assay and expression of phospho-histone γH2A.X (p-γH2A.X). Levels of cellular oxidative stress as reactive oxygen species (ROS) accumulation were measured using 2’,7’-dichlorofluorescein diacetate. The involvement of selected genes in the oxidative stress-mediated signaling pathway was explored using Western blot analysis.

**Results:**

SHME attenuated H_2_O_2_-induced growth inhibition and exhibited scavenging activity against intracellular ROS that were induced by H_2_O_2_. The SHME also inhibited comet tail formation, p-γH2A.X expression, and the number of sub-G1 hypodiploid cells, suggesting that it prevents H_2_O_2_-induced cellular DNA damage and apoptotic cell death. Furthermore, the SHME significantly enhanced the expression of heme oxygenase-1 (HO-1) associated with induction of nuclear factor-erythroid 2 related factor 2 (Nrf2) in a time- and concentration-dependent manner. Moreover, the protective effect of the SHME on H_2_O_2_-induced C2C12 cell damage was significantly abolished by zinc protoporphyrin IX, a HO-1 competitive inhibitor, in C2C12 cells.

**Conclusions:**

These findings suggest that the SHME augments cellular antioxidant defense capacity through both intrinsic free radical scavenging activity and activation of the Nrf2/HO-1 pathway, protecting C2C12 cells from H_2_O_2_-induced oxidative cytotoxicity.

## Background

Oxidative stress is defined as a disturbance in the balance between the production of reactive oxygen species (ROS) and antioxidant defenses. ROS are ions or small molecules including oxygen species, that are produced as normal products of cellular metabolism. Substantial data have shown the essential role of oxidative stress in the regulation of diverse cellular events [1,2]. Furthermore, some ROS act as cellular messengers during redox signaling. However, disturbances in the normal redox state of cells and/or a concomitant decline in antioxidant scavenging capacity can cause toxic effects through the production of peroxides and free radicals that damage all components of the cell, including proteins, lipids, and nucleic acids. Moreover, elevated production of ROS increases oxidative stress, leading to cellular dysfunction and, eventually, apoptotic cell death [3-5]. Thus, oxidative stress can cause disruptions in normal cellular signaling mechanisms.

Because ROS formation occurs naturally, mammalian cells have developed several adaptive mechanisms to limit ROS formation or to detoxify ROS. These mechanisms use antioxidant enzymes or antioxidant compounds. Among the various antioxidant enzymes, the protective role of heme oxygenase-1 (HO-1), an inducible isoform of the first and rate-limiting enzyme of heme degradation, against oxidative stress, has been emphasized [6-8]. HO-1 is regulated by the nuclear factor-erythroid 2-related factor 2 (Nrf2)-antioxidant response element (ARE) pathway, and induction of this enzyme protects cells against oxidative stress-induced cell death and tissue injury. Antioxidants are essential substances that possess the ability to protect cells from damage caused by ROS-mediated oxidative stress [9-11]. For this reason, many investigators have searched for natural antioxidants that have safe and effective pharmacological activity with low cytotoxicity and that prevent oxidative stress-mediated cellular damage.

Seaweeds have been rich sources of minerals, vitamins, and dietary fiber in East Asia for centuries [12,13]. Today, they are highlighted as multifunctional foods for maintaining health. Among them, *Sargassum horneri* (Turner) C. Agardh, an edible brown alga, is usually found in the coastal seas of Korea and Japan. Generally, *S. horneri* demonstrates antivirus [14-16], antioxidant [17,18], and anticoagulant activities [19], and preventative effects on bone loss by stimulating osteoblastic bone formation [20]. The protective actions of *S. horneri* against ultra violet (UV) A-induced damage have been reported; in particular, the chromene compound isolated from *S. horneri* shields human dermal fibroblasts from UV A-induced oxidative stress [21,22]. However, little research has been reported regarding the protective effects of *S. horneri* against oxidative stress. Thus, the aim of this study was to examine the ability of a *S. horneri* methanol extract (SHME) to protect C2C12 murine skeletal muscle cells from hydrogen peroxide (H_2_O_2_)-induced cell damage and to determine the mechanism underlying these protective effects.

## Methods

### Preparation of the SHME

The SHME (stock number AC023) was purchased from Jeju Bio-Resource Extract Bank (Jeju HI-Tech Industry Development Institute, Jeju, Republic of Korea). Briefly, fresh *S. horneri*, which was authenticated by Professor S.H. Hong, Department of Biochemistry, Dongeui University College of Oriental Medicine (Busan, Republic of Korea), collected along the Jeju Island coast of Korea in July 2005, was washed three times with tap water to remove salt, epiphytes, and sand before storage at -20°C. The frozen samples were lyophilized and homogenized using a grinder before extraction. The dried powder was extracted with 70% methanol (SHME) and evaporated *in vacuo* and dissolved in dimethyl sulfoxide (DMSO, Sigma-Aldrich Chemical Co., St. Paul, MN, USA). A voucher specimen (accession number DEU-25) was deposited at the Natural Resource Bank of Dongeui University College of Oriental Medicine.

### Cell culture and treatment

Mouse-derived C2C12 myoblasts obtained from the American Type Culture Collection (Manassas, VA, USA) were cultured in Dulbecco’s modified Eagle’s medium (DMEM, Gibco-BRL, Gaithersburg, MD, USA) supplemented with 10% heat-inactivated fetal bovine serum (FBS, Gibco-BRL), 100 U/ml penicillin G, 100 μg/ml streptomycin, and 0.25 μg/ml amphotericin fungizone at 37°C in a humidified atmosphere of 5% CO_2_ in air. The SHME was dissolved in DMSO as a stock solution at 50 mg/ml, and the stock solution was then diluted with medium to the desired concentration prior to use.

### Cell viability assay

C2C12 cells were seeded in 6-well plates at a density of 1 × 10^5^ cells per well. After a 24-h incubation, the cells were treated with various concentrations of SHME in the absence or presence of H_2_O_2_ and/or zinc protoporphyrin IX (ZnPP, Sigma-Aldrich) for the times indicated. An MTT (3-(4,5-dimetylthiazol-2-yl)-2,5-diphenyl-tetrazolium, Sigma-Aldrich) working solution was added to the culture plates and incubated for 3 h at 37°C. The culture supernatant was removed from the wells, and DMSO was added to completely dissolve the formazan crystals. The absorbance of each well was measured at 540 nm with a microplate reader (Molecular Devices, Palo Alto, CA, USA). The effect of the SHME on cell growth was assessed as the percentage of cell viability, where the vehicle-treated cells were considered 100% viable.

### Morphological imaging

Morphological changes were monitored by obtaining photomicrographs under an inverted phase contrast microscope (Carl Zeiss, Oberkochen, Germany).

### Comet assay (single-cell gel electrophoresis)

The cell suspension was mixed with 0.5% low melting agarose (LMA) at 37°C, and the mixture was spread on a fully frosted microscopic slides precoated with 1% normal melting agarose. After solidification of the agarose, the slide was covered with 0.5% LMA and then immersed in a lysis solution (2.5 M NaCl, 100 mM Na-EDTA, 10 mM Tris, 1% Triton X-100, and 10% DMSO, pH 10) for 1 h at 4°C. The slides were then placed in a gel electrophoresis apparatus containing 300 mM NaOH and 10 mM Na-EDTA (pH 13) for 40 min to allow for DNA unwinding and expression of alkali-labile damage, and then an electrical field was applied (300 mA, 25 V) for 20 min at 4°C to draw the negatively charged DNA toward the anode. After electrophoresis, the slides were washed three times for 5 min at 4°C in a neutralizing buffer (0.4 M Tris, pH 7.5), followed by staining with 20 μg/ml propidium iodide (PI, Sigma-Aldrich). The slides were examined under a fluorescence microscope (Carl Zeiss).

### Protein extraction and Western blot analysis

After removing the media, the cells were washed with ice-cold PBS and gently lysed for 20 min in ice-cold lysis buffer (40 mM Tris, pH 8.0, 120 mM, NaCl, 0.5% NP-40, 0.1 mM sodium orthovanadate, 2 μg/ml leupeptin, and 100 μg/ml phenymethylsulfonyl fluoride). The supernatants were collected and protein concentrations were determined using a Bio-Rad protein assay kit (Bio-Rad, Hercules, CA, USA). For Western blotting, equal amounts of protein extracts (typically 30 μg) were separated by denaturing SDS-polyacrylamide gel electrophoresis and transferred electrophoretically to PVDF membranes (Schleicher & Schuell, Keene, NH, USA). The membranes were incubated overnight at 4°C with primary antibodies, probed with enzyme-linked secondary antibodies (Amersham, Arlington Heights, IL, USA) for 1 h at room temperature, and detected using an enhanced chemiluminescence (ECL) detection system (Amersham). Antibodies were purchased from Santa Cruz Biotechnology (Santa Cruz, CA, USA) and Cell Signaling Technology (Danvers, MA, USA).

### Measurement of ROS

The cells were incubated with 10 μM 2’,7’-dichlorofluorescein diacetate (DCF-DA, Molecular Probes, Eugene, OR, USA) for 20 min at room temperature in the dark to monitor ROS production. ROS production in the cells was monitored with a flow cytometer (Becton Dickinson, San Jose, CA, USA) and the Cell-Quest Pro software [23].

### Detection of apoptotic sub-G1 hypodiploid cells

Harvested cells were fixed in 75% ethanol at 4°C for 30 min and incubated at room temperature for 30 min in the dark in PBS containing PI and RNase A. Sub-G1 hypodiploid cells were assessed using a flow cytometer.

### Assessment of apoptosis by flow cytometry

To assess the induced cell apoptosis rate quantitatively, a fluorescein-conjugated Annexin V (Annexin V-FITC) staining assay was performed according to the manufacturer’s protocol (BD Biosciences Pharmingen, San Jose, CA, USA). Briefly, cells were stained with 5 μl Annexin V-FITC and 5 μl PI in each sample. After incubation for 15 min at room temperature in the dark, the degree of apoptosis was quantified as a percentage of the Annexin V-positive and PI-negative cells by flow cytometry.

### Statistical analysis

Data are expressed as means ± standard error of the mean (SEM). The results were subjected to an analysis of variance using Tukey’s test to analyze differences. A *p* value < 0.05 was considered to indicate statistical significance.

## Results

### SHME increases the viability of H_2_O_2_-treated C2C12 cells

**Figure 1:**
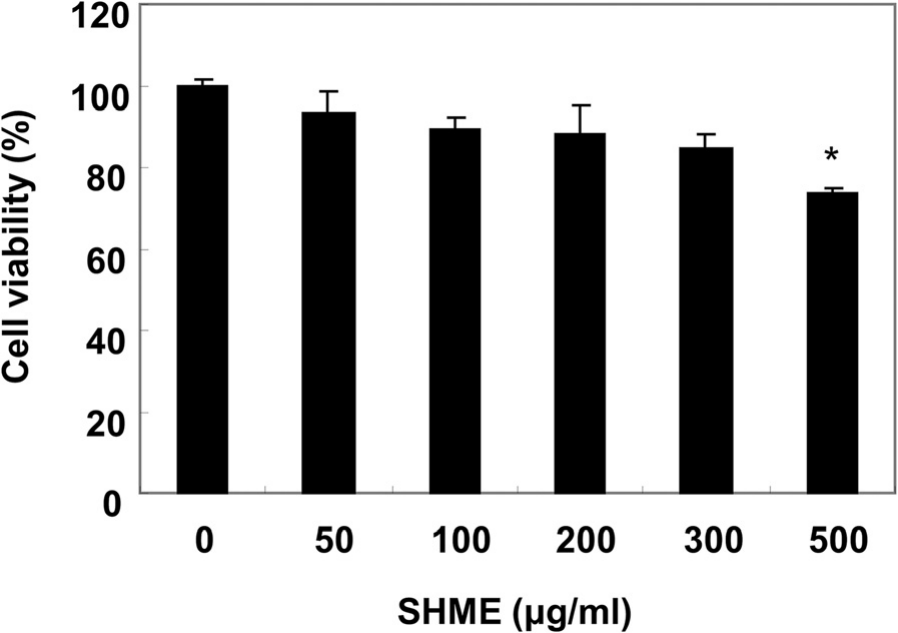
**Effect of SHME on viability of C2C12 cells.** C2C12 cells were incubated for 24 h with various concentrations of SHME for 24 h. Cell viability was estimated by the MTT assay. Data are presented as the mean ± SEM obtained from three independent experiments (*P < 0.05, compared with the control group).

**Figure 2:**
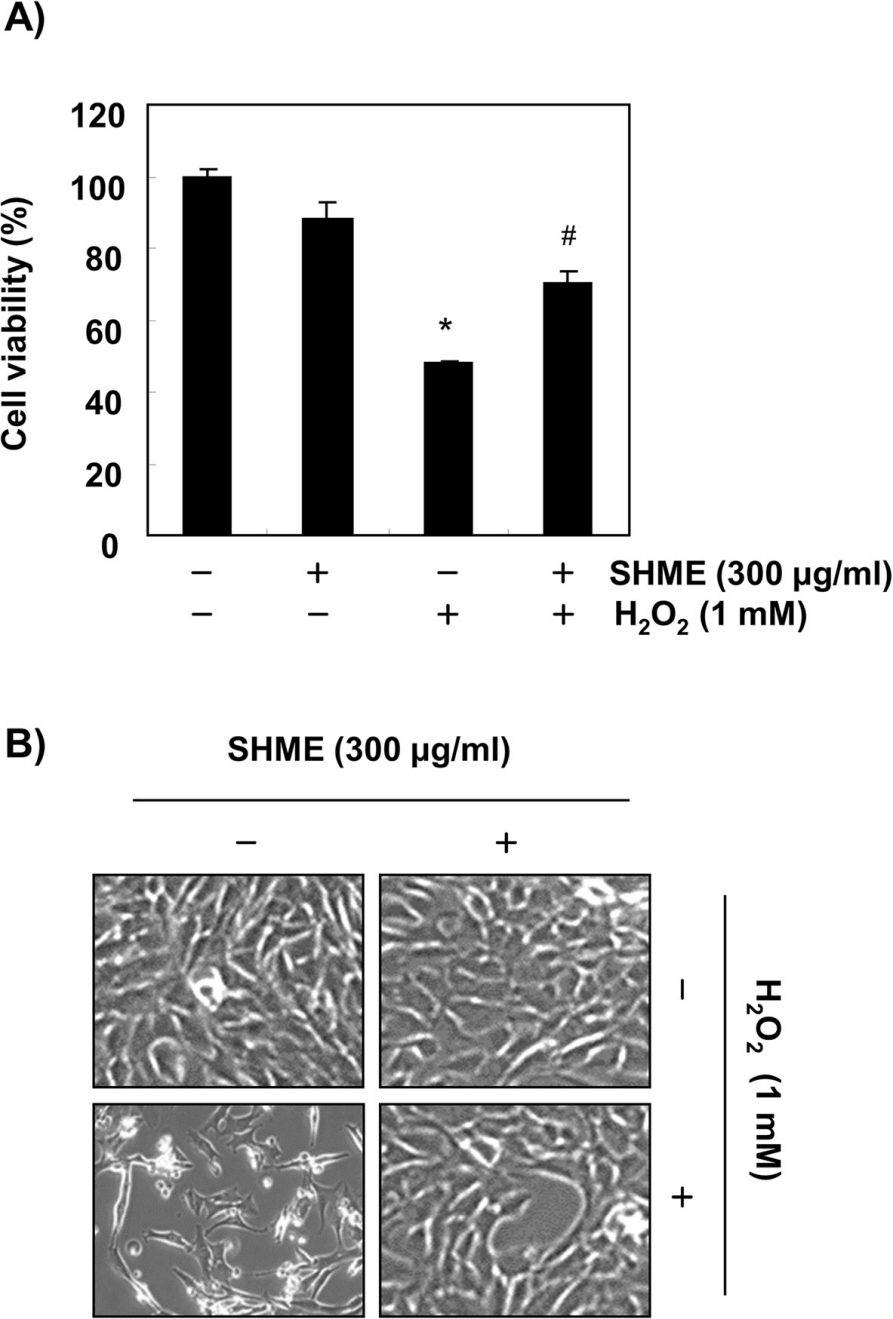
**Effect of SHME on H**_**2**_ **O**_**2**_ **-induced growth inhibition and morphological changes in C2C12 cells.** C2C12 cells were pretreated with 300 μg/ml SHME for 1 h and then incubated with or without 1 mM H_2_O_2_ for 6 h. Then, cell viability **(A)** and changes in cell morphology **(B)** were measured. Data are presented as the mean ± SEM obtained from three independent experiments (*P < 0.05, compared with the control group; ^#^ *P* < 0.05, compared with the H_2_O_2_-treated group).

C2C12 cells were stimulated with varying concentrations of the SHME for 24 h and their viability was measured using the MTT assay to evaluate the cytotoxic potential of the SHME. SHME alone at 50-300 μg/ml showed no cytotoxic effects (Figure 1), but significant cytotoxicity was seen at 500 μg/ml SHME. Thus, 300 μg/ml SHME was chosen as the optimal dose for studying the cytoprotective effect of SHME against H_2_O_2_-induced cell damage. Cell viability was assessed again to investigate whether the SHME augmented the viability of C2C12 cells exposed to 1 mM H_2_O_2_. As shown in Figure 2A, cell viability was reduced to 48.5% in H_2_O_2_-treated cells in the absence of the SHME; however, cell viability increased to 72% in H_2_O_2_-treated cells treated with 300 μg/ml SHME. In addition, H_2_O_2_ stimulation induced significant morphological changes, which were effectively attenuated by SHME pretreatment (Figure 2B).

### SHME attenuates H_2_O_2_-induced apoptosis in C2C12 cells

**Figure 3:**
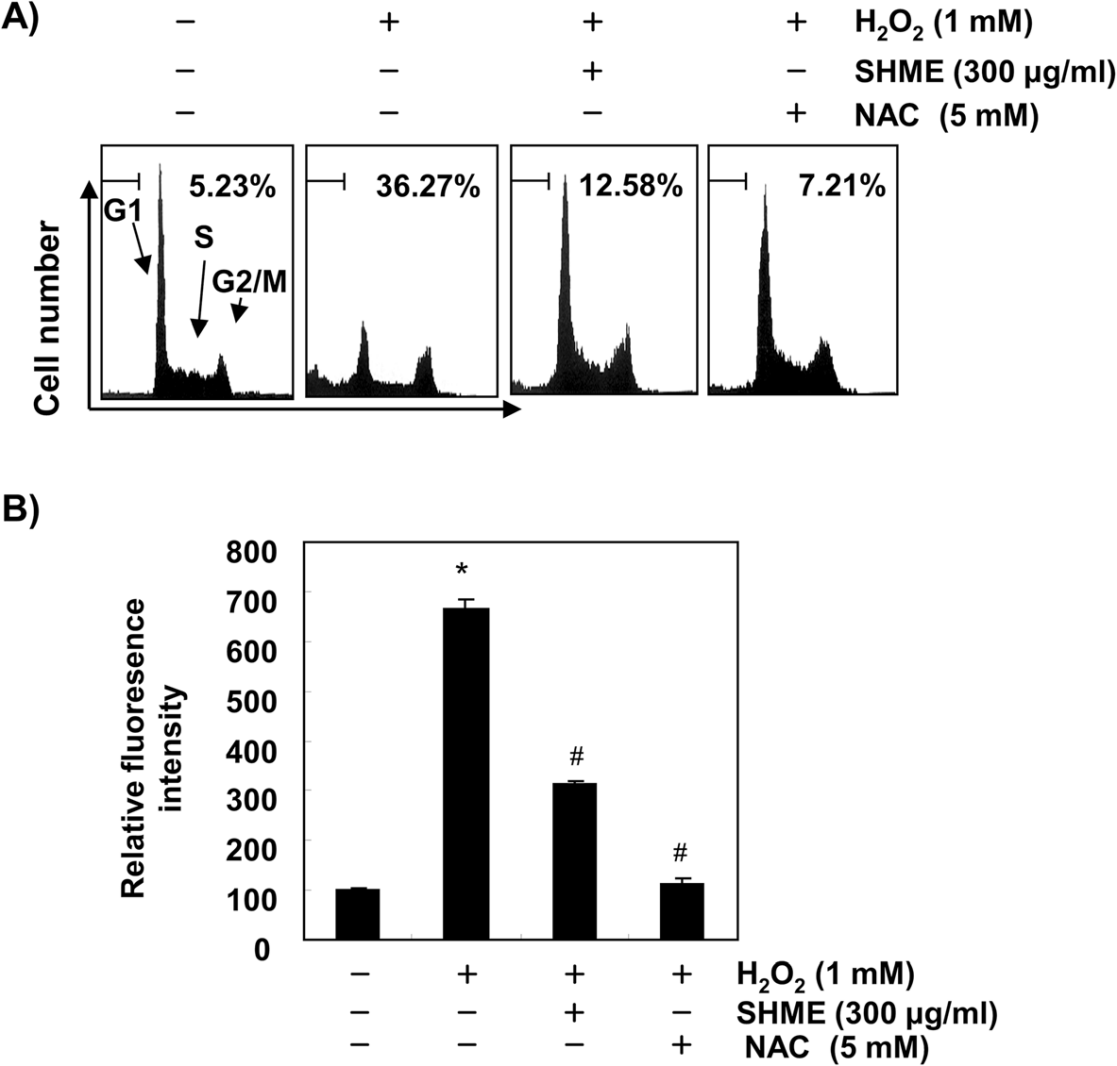
**SHME attenuates H**_**2**_ **O**_**2**_ **-induced apoptosis and ROS generation in C2C12 cells.** C2C12 cells were pretreated with 300 μg/ml SHME or 5 mM NAC for 1 h and then stimulated with and without 1 mM H_2_O_2_ for 6 h. **(A)** To quantify the degree of apoptosis, media were discarded and the cells were evaluated for sub-G1 DNA content using a flow cytometer. **(B)** To monitor ROS production, the cells were incubated at 37°C in the dark for 20 min with new culture media containing 10 μM DCF-DA. ROS generation was measured using a flow cytometer. Data are presented as the mean ± SEM obtained from three independent experiments (*P < 0.05, compared with the control group; ^#^ *P* < 0.05, compared with the H_2_O_2_-treated group).

To investigate the protective effect of the SHME on H_2_O_2_-induced apoptosis, the frequency of apoptotic sub-G1 cells was detected by flow cytometry. As shown in Figure 3A, C2C12 cells stimulated with H_2_O_2_ resulted in upregulation of the apoptosis ratio; however, the enhanced apoptosis ratio was significantly alleviated by preincubation with SHME. The scavenging effect of the SHME against H_2_O_2_-induced ROS was examined next using the DCF-DA assay. Our results indicated that ROS levels increased in H_2_O_2_-treated cells compared with those in untreated cells. The SHME decreased fluorescence of the DCF product, an indication of ROS generation, produced from DCF-DA by ROS in H_2_O_2_-treated cells (Figure 3B). As a positive control, the ROS scavenger N-acetyl-L-cysteine (NAC, 5 mM) also attenuated H_2_O_2_-induced apoptotic capacity and ROS generation, indicating that the SHME scavenged H_2_O_2_-induced ROS.

### SHME reduces H_2_O_2_-mediated DNA damage

**Figure 4:**
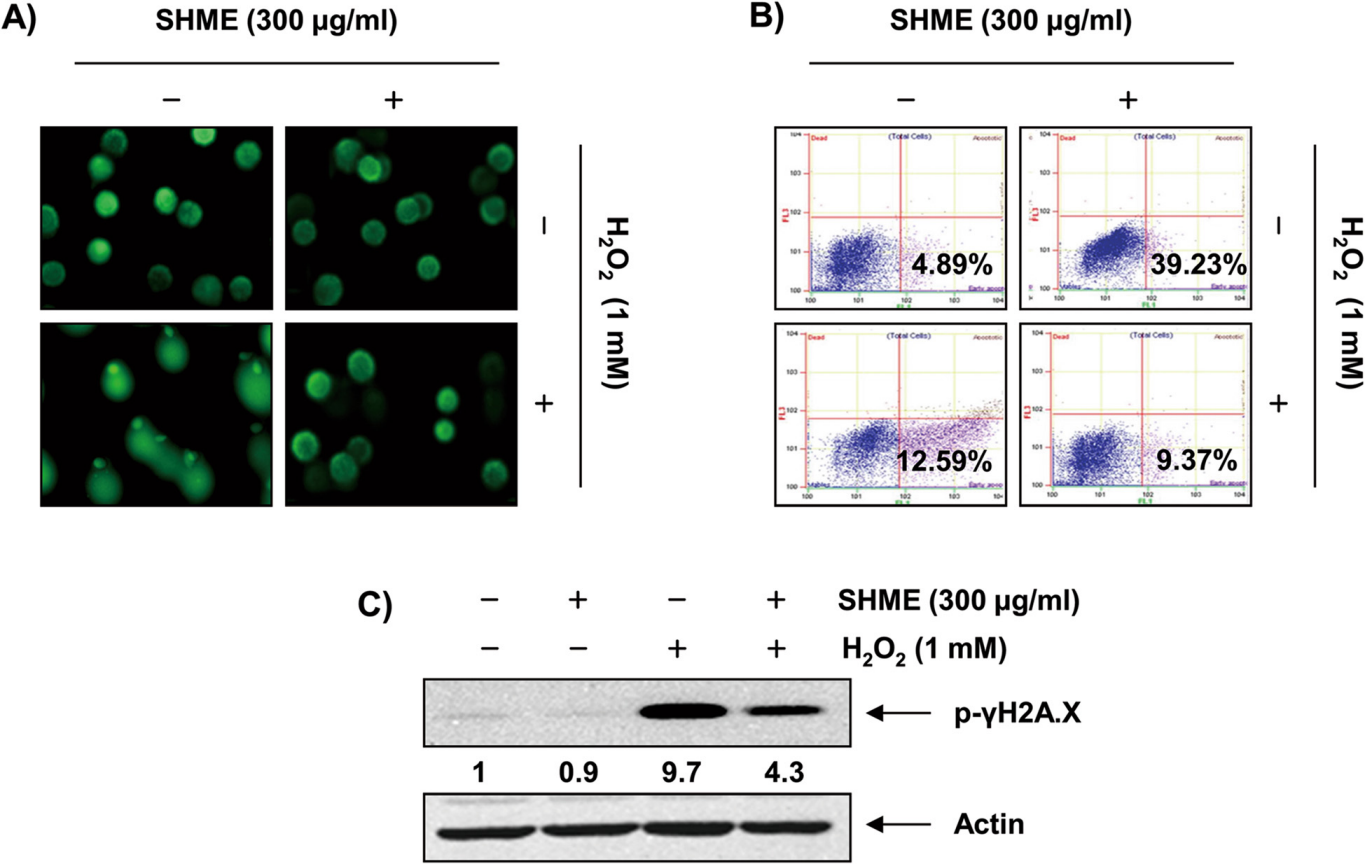
**SHME protects against H**_**2**_ **O**_**2**_ **-induced DNA damage in C2C12 cells.** C2C12 cells were pretreated with 300 μg/ml SHME for 1 h and then incubated with and without 1 mM H_2_O_2_ for 6 h. **(A)** To detect cellular DNA damage, the comet assay was performed and representative pictures of the comets were taken using a fluorescence microscope at × 200 original magnification. **(B)** To quantify the degree of apoptosis, the cells were stained with Annexin V, and the percentages of apoptotic cells were then analyzed using flow cytometric analysis. Each point represents the means of two independent experiments. **(C)** Whole-cell lysates were prepared and subjected to Western blot analysis with a specific antibody against phospho-histone γH2A.X. Actin was used as the loading control. A representative blot from three independent experiments is shown. The numbers represent the average densitometric analyses as compared with actin in, at a minimum, two or three different experiments.

We next examined H_2_O_2_-mediated damage to C2C12 cell DNA using the alkaline comet assay and Western blotting analysis. Figure 4A indicates that a longer comet tail moment (DNA migration) occurred due to an increase in H_2_O_2_-treated cells, and untreated control cells only showed typical representative nuclei. To determine whether the cytoprotective effect of SHME involved the inhibition of apoptosis induced by H_2_O_2_, we also stained cells for Annexin V/PI and assessed them by flow cytometry. We found that the percentages of apoptotic cells increased from ~4.89 to 39.23% after treatment with H_2_O_2_ (Figure 4B). However, there was a significant reduction in H_2_O_2_-treated cells pre-treated for 1 h with SHME. Additionally, our results showed that treating C2C12 cells with H_2_O_2_ resulted in the upregulation of the level of phosphorylated histone H2A.X (Ser139) (p-γH2A.X), a classic marker of DNA double-strand breaks (DSBs) (Figure 4C). However, pretreatment with SHME resulted in a significant decrease in the number of comet tails and decreased p-γH2A.X expression, indicating a protective effect of the SHME against H_2_O_2_-induced DNA damage.

### SHME upregulates HO-1 and Nrf2 protein expression

**Figure 5:**
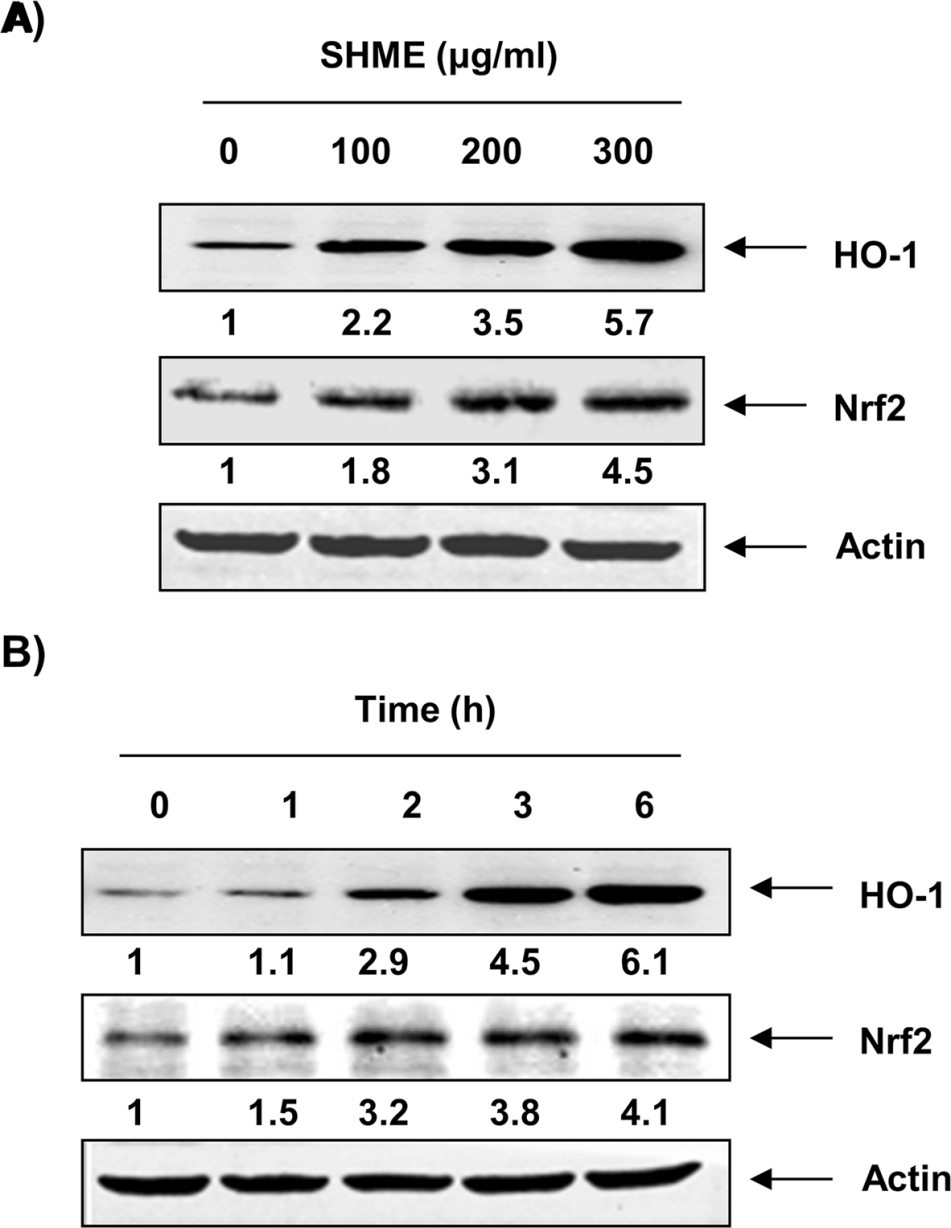
**Induction of HO-1 and Nrf2 expression by SHME in C2C12 cells.** Cells were incubated with various concentrations of the SHME for 6 h **(A)** or for the indicated periods with 300 μg/mL SHME **(B)**. The levels of HO-1 and Nrf2 proteins were determined by Western blot analyses, and representative blots of three independent experiments are shown. Actin was used as a loading control. The numbers represent the average densitometric analyses as compared with actin in, at a minimum, two or three different experiments.

Because HO-1 is an important component of cellular defenses against oxidative stress, we assessed whether non-cytotoxic concentrations of SHME would affect HO-1 protein expression. As shown in Figure 5, C2C12 cells exposed to the SHME showed concentration- and time-dependent increases in HO-1 expression compared with that in the control group. Several studies have reported that Nrf2 is an important upstream contributor to the mechanism of HO-1 expression; thus, we examined whether the SHME could induce Nrf2 expression in C2C12 cells. After exposure to SHME, C2C12 cells showed a gradual increase in Nrf2 levels, which was strongly correlated with the increase in HO-1 expression (Figure 5).

### HO-1 is involved in SHME protection against H_2_O_2_ treatment

**Figure 6:**
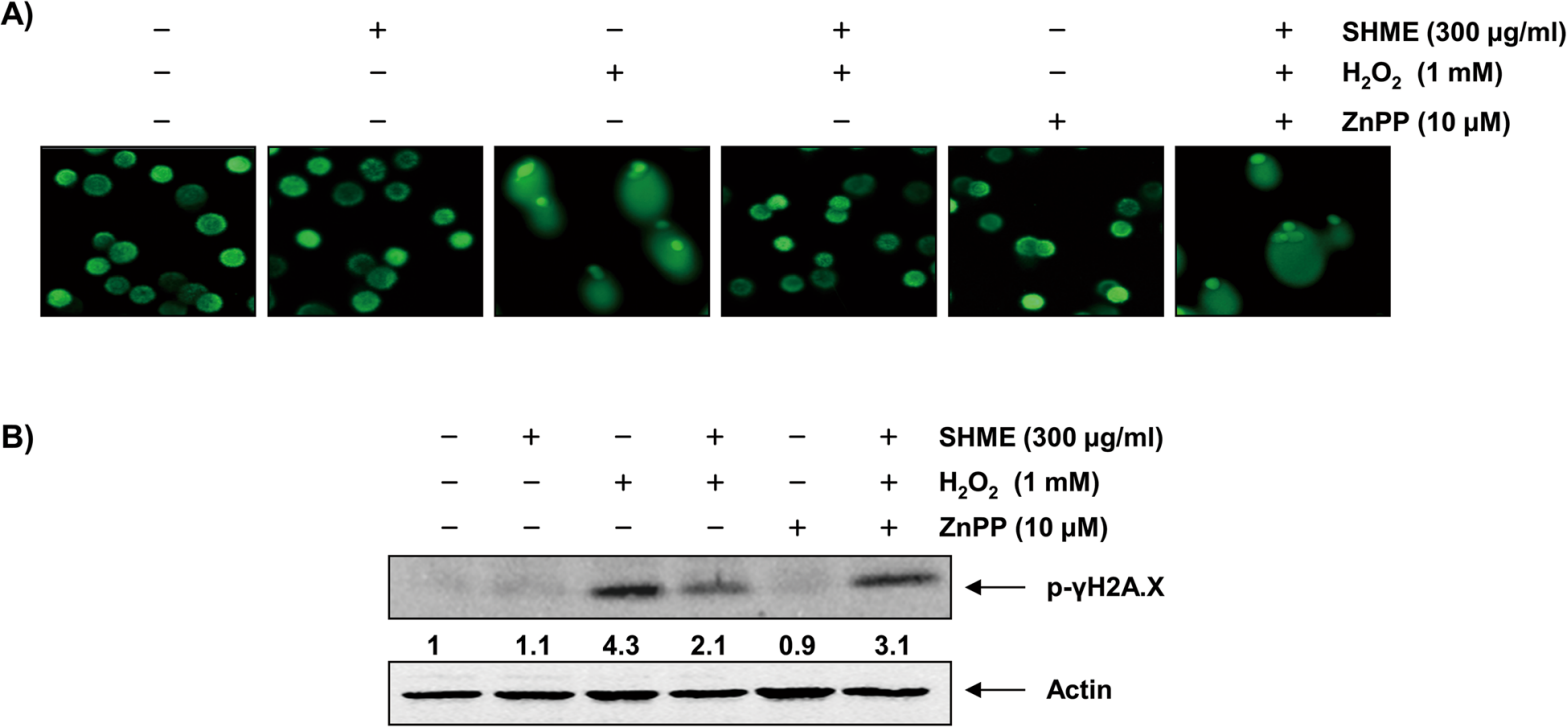
**Effects of an inhibitor of HO-1 on SHME-mediated protection of DNA damage by H**_**2**_ **O**_**2**_ **in C2C12 cells.** C2C12 cells were pretreated for 1 h with 300 μg/ml SHME and then treated for 6 h with or without 1 mM H_2_O_2_ in the absence or presence of 10 μM ZnPP. **(A)** The comet assay was performed and representative pictures of the comets were taken using a fluorescence microscope at × 200 original magnification. **(B)** Cell lysates were prepared and subjected to Western blot analysis with a specific antibody against phospho-histone γH2A.X. Actin was used as a loading control. A representative blot from three independent experiments is shown. The numbers represent the average densitometric analyses as compared with actin in, at a minimum, two or three different experiments.

Recent reports have described that elevated intracellular ROS levels lead to cellular dysfunction. Upregulation of HO-1 expression in a wide variety of cells play an important role in protection against toxicity caused by oxidative insults [6-8]. Thus, to investigate whether the protective effect of SHME is related to its inductive effect on HO-1 expression, we blocked HO-1 activity using ZnPP, a selective HO-1 inhibitor. As shown in Figure 6, the protective effect of the SHME against H_2_O_2_-induced DNA damage was hindered by ZnPP.

**Figure 7:**
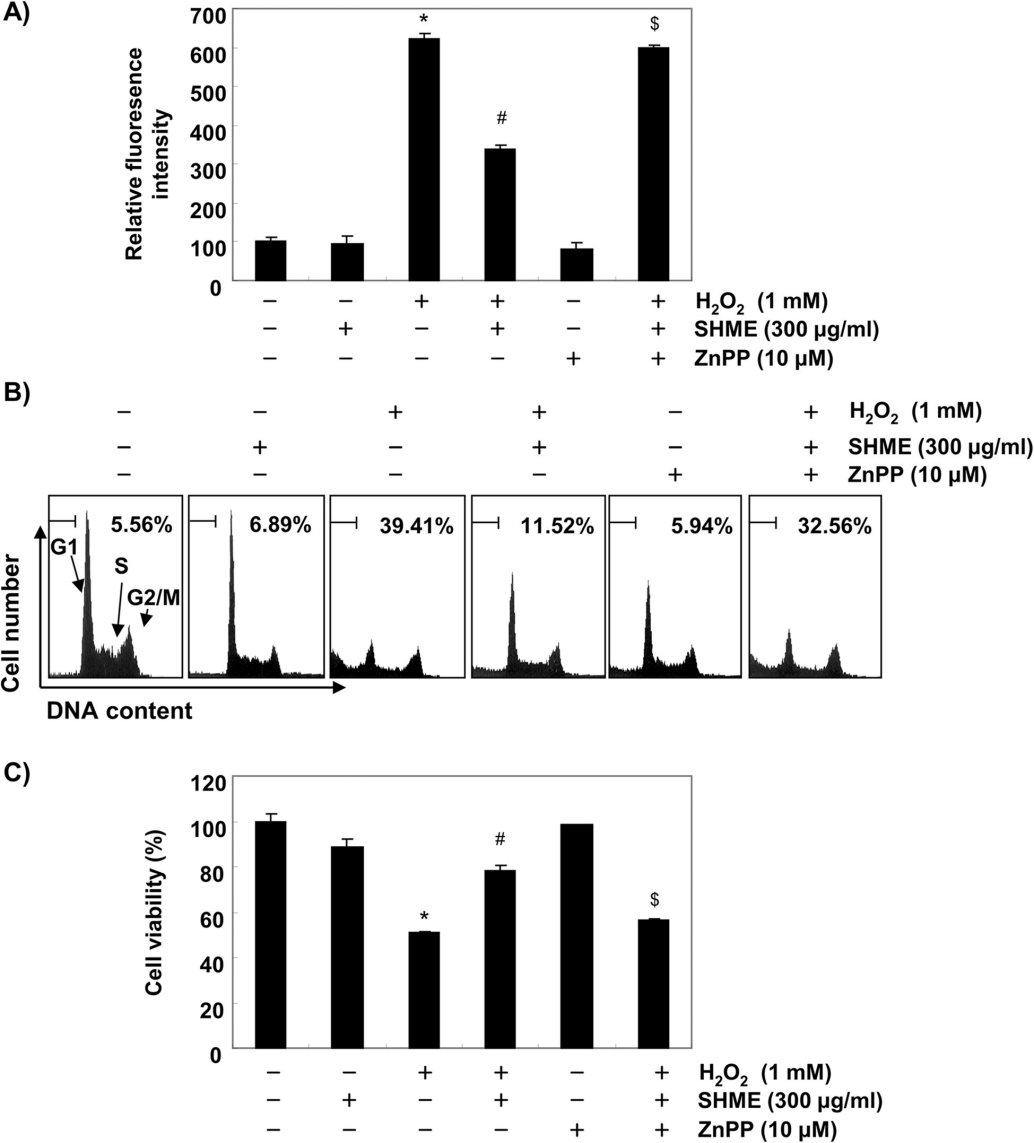
**Effects of an inhibitor of HO-1 on SHME-mediated attenuation of ROS formation and apoptosis induction by H**_**2**_ **O**_**2**_ **in C2C12 cells. (A)** Cells grown under the same conditions as those in Figure 6 were assayed for ROS generation by DCF fluorescence. **(B)** The degree of apoptosis was evaluated by sub-G1 DNA content using a flow cytometer. **(C)** Cell viability was estimated by the MTT assay. Data are presented as the mean ± SEM, obtained from three independent experiments (*P < 0.05, compared with the control group; ^#^ *P* < 0.05, compared with the H_2_O_2_-treated group; ^$^ *P* < 0.05, compared with the H_2_O_2_ and SHME-treated group).

Furthermore, ZnPP significantly reversed the inhibition of ROS generation and apoptotic activity by SHME in H_2_O_2_-stimulated C2C12 cells (Figure 7A, B). In contrast, we also found that ZnPP completely abolished restoration of cell viability by SHME in H_2_O_2_-treated C2C12 cells (Figure 7C). These results suggest that induction of HO-1 expression by the SHME is involved in reducing intracellular ROS levels and the cytotoxicity induced by H_2_O_2_, which leads to SHME-dependent protection from oxidative stress.

## Discussion

The antioxidative effects and bioactivities of several different crude extracts and certain components of various marine algae have been evaluated both *in vitro* and *in vivo*. Because oxidative stress plays important roles in the occurrence and progress of various human diseases, natural marine algal products are attractive targets for the development of novel health-promoting agents [12,13]. In this study, as part of our screening program for therapeutic antioxidative agents from seaweeds, we investigated whether SHME, a methanol extract derived from *S. horneri*, had protective effects against H_2_O_2_-induced cytotoxicity in C2C12 cells.

Generally, mild ROS conditions stimulate cellular antioxidant systems, which protect against oxidative stress, but extreme ROS can destroy the cytoprotective defense mechanisms, such as antioxidant and DNA repair systems. Such oxidative damage to cells ultimately leads to cell death, including programmed cell death or apoptosis [9-11]. In the present study, C2C12 cells exposed to H_2_O_2_ exhibited significantly decreased cell viability and increased apoptosis. Notably, SHME increased cell viability significantly by inhibiting H_2_O_2_-induced apoptosis and reduced ROS generation generated by H_2_O_2_ treatment in C2C12 cells (Figures 2 and 3). Additionally, H_2_O_2_ treatment increased the expression of p-γH2A.X, an indicator of DSB formation [24], and DNA tail length in the comet assay, whereas each event was mitigated in C2C12 cells by treatment with SHME prior to H_2_O_2_ exposure (Figure 4). These results suggest that the SHME protected cellular DNA against oxidative stress damage.

Among the various antioxidant/cytoprotective enzymes, HO-1 has received considerable attention. HO-1 is readily induced in response to oxidative stress, and the induction of HO-1 results in a relatively higher resistance to oxidative damage. Transcriptional regulation of the HO-1 gene is linked to the transcription factor Nrf2, which plays a key role in cellular defenses [9-11]. Nrf2 is localized in an inactive form in the cytoplasm, where it is anchored by its inhibitor protein, Kelch-like ECH-associated protein 1 (Keap1). In response to oxidative stress, Nrf2 is released from Keap1 and transmits a stress signal to the nucleus for the activation of a distinct set of genes encoding phase II detoxifying enzymes as well as several stress responsive proteins, including HO-1 [7,8]. Therefore, we were further interested in determining the potential role of HO-1 in H_2_O_2_-induced C2C12 cell damage and SHME-mediated cytoprotection. We have provided evidence for the induction of HO-1 by SHME and showed that SHME-induced HO-1 protein expression occurred in a concentration- and time-dependent manner, with a concomitant increase in Nrf2 expression (Figure 5). We further confirmed that exogenous induction of HO-1 by SHME was useful in H_2_O_2_-induced oxidative damage of C2C12 cells. Our data indicate that inhibiting HO-1 function using the HO-1 inhibitor ZnPP effectively reduced the protective effect of the SHME against H_2_O_2_-induced DNA damage (Figure 6) as well as cytoprotection and ROS generation (Figure 7). The present results demonstrate that the induction of HO-1 by SHME was responsible for protecting C2C12 cells against H_2_O_2_-induced oxidative stress, consistent with other studies showing that HO-1 plays an important role protecting cells against oxidative stress [25,26]. The results also suggest that SHME-induced cytoprotection of C2C12 cells against oxidative stress was critically dependent on activation of the Nrf2/HO-1 pathway.

## Conclusions

Taken together, our results suggest that SHME may have multiple mechanisms of action against oxidative damage that affect cytoprotection both by reducing ROS generation and boosting HO-1 induction for ROS detoxification. These results support the potential therapeutic mechanism of SHME in protecting against oxidative stress-related diseases with additional research using experimental *in vivo* disease models.
